# Simple economics of vaccination: public policies and incentives

**DOI:** 10.1007/s10754-024-09367-2

**Published:** 2024-03-22

**Authors:** Jesús Villota-Miranda, R. Rodríguez-Ibeas

**Affiliations:** Department of Economics, University of La Rioja, La Cigüeña 60, 26004 Logroño, Spain

**Keywords:** Vaccination, Herd immunity, SIR model, Public policies, Endemic steady state, I11, I12

## Abstract

This paper focuses on the economics of vaccination and, more specifically, analyzes the vaccination decision of individuals using a game-theoretic model combined with an epidemiological SIR model that reproduces the infection dynamics of a generic disease. We characterize the equilibrium individual vaccination rate, and we show that it is below the rate compatible with herd immunity due to the existence of externalities that individuals do not internalize when they decide on vaccination. In addition, we analyze three public policies consisting of informational campaigns to reduce the disutility of vaccination, monetary payments to vaccinated individuals and measures to increase the disutility of non-vaccination. If the public authority uses only one type of policy, herd immunity is not necessarily achieved unless monetary incentives are used. When the public authority is not limited to use only one policy, we find that the optimal public policy should consist only of informational campaigns if they are sufficiently effective, or a combination of informational campaigns and monetary incentives otherwise. Surprisingly, the requirement of vaccine passports or other restrictions on the non-vaccinated are not desirable.

## Introduction

Herd immunity is the minimum vaccination level that eradicates an infectious disease, but the level is difficult to achieve without policy incentives. Vaccination is an activity that generates positive and negative externalities, but individuals do not usually internalize them. As a result, the vaccination level is too low, and it is common to see the adoption of public policies to change the perceived benefits and costs of vaccination and non-vaccination and incentivize individual vaccination decisions. For instance, the European Centre for Disease Prevention and Control in its technical report “*Facilitating COVID-19 vaccination acceptance and uptake in the EU/EEA*” mentions some public policies that were implemented during the COVID-19 pandemic by European countries to facilitate the acceptance and adoption of vaccination against COVID-19. The Irish Health Department launched “SciComm”, an initiative in which a network of scientists promoted confidence in the vaccination among young people by answering their queries. Likewise, in Spain, the #YoMeVacunoSeguro campaign highlighted the benefits of vaccination. In Serbia, the government paid 25€ to each vaccinated individual (Savalescu et al., 2021). A study carried out in Sweden in 2021 on 8286 participants showed that vaccination rates increased 4.2% if vaccinated individual were paid 24 USD (Campos-Mercade et al. 2021). In 2021, the European Union established the EU Digital COVID Certificate to facilitate safe travel across Member States. Likewise, lotteries awarding important prizes to winners if vaccinated were held in some US states (Savalescu et al., 2021). In Australia, the initiative "*No Jab, No Pay*" withheld state payments for parents of children under 20 years of age who are not vaccinated (Savalescu et al., 2021). Similarly, the policy “No Jab, No Play” did not allow unvaccinated children to attend preschool and childcare centres (Savalescu et al., 2021). In this paper, we follow Bauch and Earn (2004) to characterize first the individual vaccination decision considering the vaccination and non-vaccination costs.^1^ We focus next on the design of public policies that incentivize vaccination. In particular, we focus on three types of policies: informational campaigns to reduce the perceived vaccination costs, policies based on monetary payments to vaccinated individuals, and policies to increase the costs borne by non-vaccinated individuals (for instance, vaccine passports), and characterize the optimal intervention public policy.

The economic analysis of vaccination has a strategic decision component, framed within the perspective of game theory, and an epidemiological component, which describes the dynamics of the disease and characterizes the probability of transmission. Chang et al. (2020) provide a complete review of the literature and describe the different approaches followed to analyze the problem of vaccination. The epidemiological models used in the literature are of two types: compartmental models (Brauer, 2008; Perisic & Bauch, 2009a, 2009b) and network models (Craft & Caillaud, 2011; Lloyd & Valeika, 2007; Neilson & Xiao, 2018). The most classical compartmental model is known as the SIR model, developed by Kermack and McKendrick in (1927). Numerous modifications have built on this pioneering model to adapt it to the epidemiological dynamics of each particular disease (see, for example, Bauch & Earn, 2004; Lim & Zhang, 2017a, 2017b; Rusu, 2015). On the other hand, the strategic models often ignore the benefits of vaccination, and include only the costs or disutilities. Disutilities have been defined in terms of monetary value (Galvani et al., 2007; Sorensen, 2023; Yamin et al., 2014); in terms of risk (Bauch, 2005; Bauch & Earn, 2004; Bauch et al., 2003; De Donder et al., 2021; Liu et al., 2012), or using other indicators (as Basu et al., 2008, who used QALYs).

Most models use non-cooperative games of complete information, assuming that individuals are rational and always choose the strategy that maximizes their effective utility, and characterize the Nash equilibrium strategy profile. Due to the positive externality generated by vaccination, the Nash equilibrium is affected by “free-riding” behavior, and it is not possible to eradicate the disease with voluntary vaccination. However, other studies reach a different conclusion (De Donder et al., 2021; Lim & Zhang, 2017a, 2017b; Perisic & Bauch, 2009b).^2^ Some papers analyze the use of public policies to improve vaccination rates. Gans (2023) focuses on how the prevalence on the disease may affect vaccine hesitancy, and analyzes the impact of public policies to reduce it.

In particular, the paper focuses on the use of vaccine passports, and finds surprisingly they can reduce the prevalence of the disease, and increase hesitancy. Iyer et al. (2022) conduct an online randomized experiment on a sample of 2461 individual to analyze the effectiveness of informational campaigns and monetary incentives to reduce vaccine hesitancy. They found that a 1,000USD incentive increases vaccination rate up to 86.9%.

Our analysis follows the most used approach in the literature and consider a strategic game of complete information together with an epidemiological SIR model to analyze the use of the aforementioned public policies to increase vaccination rates. As in the literature, we find that the equilibrium individual vaccination rate is below the rate compatible with herd immunity. When public authorities use only one type of policy, we find that herd immunity is not necessarily achieved with informational campaigns or with restrictions on unvaccinated individuals. However, herd immunity is reached when monetary incentives are used. When public authorities are not restricted to use only one policy and all policy tools are available, we find that the optimal public policy should consist only of informational campaigns if they are sufficiently effective, or a combination of informational campaigns and monetary incentives otherwise. In both cases, herd immunity is achieved.

Intuitively, the result is driven by the distinct nature of the two types of interventions. On the one hand, the benefits of an informational campaign have the characteristics of a public good: *non-rivalry and non-excludability.* The benefits of the campaign reach the entire population and the positive effects enjoyed by one individual do not reduce the benefits available to others. On the contrary, monetary incentives have the characteristics of private goods (*rivalry and excludability*). The money received by a vaccinated individual is not available to another individual, and non-vaccinated individuals are excluded unless they decide to get vaccinated. If the informational campaign is effective in the sense that it convincingly reaches the majority of the population, the public authority will prefer to use this type of intervention rather than one based on monetary incentives, since it achieves the same goal (herd immunity) at a lower cost. However, if the informational campaign is not sufficiently effective, it will have to be complemented with monetary incentives. Surprisingly, policies to increase the costs borne by non-vaccinated individuals are not desirable in the optimal policy package. This finding is contradictory to real-world policies such as vaccination requirements for school or vaccine passports for cross-border travelers.

We structure the paper as follows. In section "The model", we describe the model, considering the vaccination game and the epidemiological model that describe the evolution of the disease, and characterize the Nash equilibrium. In section "The social cost of the disease", we introduce the social costs of the disease and analyze each public policy. In section "The optimal policy to promote vaccination", we characterize the optimal public policy. Finally, Section "Conclusions" presents the conclusions of the analysis, as well as some of its limitations.

## The model

### The vaccination game

Following Bauch and Earn (2004), we consider a vaccination game where individuals have to decide whether to be vaccinated when faced with an infectious disease. Let a population be composed of $n$ identical individuals each of which chooses the vaccination probability ${p}_{i}\in [\mathrm{0,1}]$, $i=1,..,n$. As individuals are identical, ${p}_{i}=p$ for all $i=1,\dots ,n$, and the population vaccination rate is $p$. Each individual decision depends on the benefits and costs of vaccination and non-vaccination. Let ${D}_{I}>0$ be the cost that an individual perceives if he/she becomes infected. This cost includes the cost related to the symptoms of the disease and to possible hospitalizations. Let ${D}_{S}\ge 0$ be the cost individual bears from the restrictions that public authorities may impose on unvaccinated individual (*e.g.*, vaccination certificates required to access some public and private venues, and even economic sanctions). Let $\phi \left(p\right)$ be the probability of infection of an unvaccinated individual when the population vaccination rate is $p$. The expected disutility or cost if the individual does not get vaccinated when the population vaccination rate is $p$ is given by:

$$\mathcal{D}\left(\text{non-vaccination; }p\right)= \phi \left(p\right){D}_{I}+{D}_{S}$$

Let ${D}_{V}>0$ be the economic cost if the individual gets vaccinated. This cost may include the value of the time required to go to the vaccination center, the pain of the syringe, and the adverse effects of the vaccine. Let $r\in (\mathrm{0,1})$ denote the efficacy of the vaccine. The probability of infection of a vaccinated individual is $\left(1-r\right)\phi \left(p\right)$. The expected disutility or cost if the individual gets vaccinated when the population vaccination rate is $p$ is given by:

$$\mathcal{D}\left(\text{vaccination; }p\right)={D}_{V}+\left(1-r\right)\phi \left(p\right){D}_{I}$$

We assume that ${D}_{I}>{D}_{V}>{D}_{S}$. It seems plausible to assume for the individuals that the perceived costs if they become infected are higher than the perceived costs if they are vaccinated. We also assume ${D}_{V}-{D}_{S}<r{D}_{I}$. Otherwise, the characterization of the equilibrium strategies is trivial as no one would get vaccinated. Each individual chooses the strategy that minimizes his/her cost or disutility. The strategy profile chosen by the individuals must be a Nash equilibrium.

#### Proposition 1

The equilibrium vaccination probability ${p}^{*}$ is:

$$p^{*} = \left\{ {\begin{array}{*{20}l} 0 \hfill & {if\;\phi \left( 0 \right) \le \frac{{D_{V} - D_{S} }}{{rD_{I} }}} \hfill \\ {\phi^{ - 1} \left( {\frac{{D_{V} - D_{S} }}{{rD_{I} }}} \right)} \hfill & {if\;\phi \left( 0 \right) > \frac{{D_{V} - D_{S} }}{{rD_{I} }}} \hfill \\ \end{array} } \right.$$

#### Proof

See Appendix.

### The epidemiological model

To find the equilibrium vaccination probability, we need to know the function $\phi \left(p\right)$. In other words, we need to specify the epidemiological model that describes the transmission dynamics of the disease. Following Bauch and Earn (2004), we consider a deterministic SIR epidemiological model where the susceptible individuals in a population interact with each other and are exposed to an infectious disease. Once infected, the individual moves from the susceptible state (S) to the infected one (I). After infection, individual may recover and move to the recovered state (R), where he/she remains as he/she is now naturally immunized.

Let $S(t)$, $I(t)$ and $R(t)$ be the proportions of susceptible, infected and recovered individuals, respectively, at time $t$. The dynamics of the three population subgroups is defined by the system of differential equations:

1 $$\frac{dS}{{dt}} = \mu \left( {1 - p} \right) - \beta SI - \mu S$$

2 $$\frac{dI}{{dt}} = \beta SI - \gamma I - \mu I$$

3 $$\frac{dR}{{dt}} = \mu p + \gamma I - \mu R$$

where $\beta$ is the transmission rate, $\gamma$ is the probability of recovery ($1/\gamma$ is the mean infectious period), $\mu$ is the birth and death rate ($1/\mu$ is life expectancy), and $p$ denotes the vaccination rate of the population. Equation (1) specifies the rate of change in the proportion of susceptible individuals as the difference between the inflow of unvaccinated individuals ($\mu \left(1-p\right))$ and the outflow of susceptible individuals who become infected or die for reasons unrelated to the disease ($\beta SI+\mu S)$. Equation (2) specifies the rate of change of the proportion of infected individuals as the difference between susceptible individuals becoming infected ($\beta SI$) and the outflow of recovered and dead individuals ($\gamma I+\mu I)$. Finally, Eq. (3) specifies the rate of change in the proportion of recovered individuals as the difference between the inflow of vaccinated and recovered individuals ($\mu p+\gamma I)$ and the outflow of dead recovered individuals $(\mu R)$. The third equation of the SIR model is superfluous as, in any time period, $S(t)+I(t)+R(t)=1$.

If we measure time in units of the mean infectious period, $z=t\gamma$, Eqs. (1) and (2) can be rewritten as follows^3^:

4 $$\frac{dS}{{dz}} = f\left( {1 - p} \right) - R_{0} \left( {1 + f} \right)SI - fS$$

5 $$\frac{dI}{{dz}} = R_{0} \left( {1 + f} \right)SI - \left( {1 + f} \right)I$$

where $f=\frac{\mu }{\gamma }$ represents the infection period as a fraction of the mean lifetime and ${R}_{0}=\beta /\left(\gamma +\mu \right)>1$ is the basic reproductive ratio (*i.e.,* the number of secondary cases produced by an infected primary case in a fully susceptible population). Finally, ${R}_{0}\left(1+f\right)=\beta /\gamma$ denotes the transmission rate per mean infection period. The next proposition characterizes the two steady states to which the system converges.

#### Proposition 2

The system converges to a steady state in which the disease is endemic if the population vaccination rate $p$ is below a threshold value ${p}_{crit }=1-\frac{1}{{R}_{0}}$. The proportion of susceptible individuals $S$ is $\frac{1}{{R}_{0}}$ and the proportion of infected individuals $I$ is $\frac{f\left[{R}_{0}\left(1-p\right)-1\right]}{{R}_{0}\left(1+f\right)}$. Otherwise, the system reaches herd immunity and the disease is eradicated.

#### Proof

See Appendix.

### The Nash equilibrium

To find the equilibrium vaccination probability ${p}^{*}$, we need to know $\phi \left(p\right)$, the probability of infection of an unvaccinated individual in the steady state in which the disease is endemic. Note that such probability is 0 when the disease is eradicated. The probability $\phi \left(p\right)$ is defined as the proportion of infected individuals over the whole population: $\frac{\beta SI}{\beta SI+\mu S}=\frac{{R}_{0}\left(1+f\right)SI}{{R}_{0}\left(1+f\right)SI+fS}$. Considering the values for $S$ and $I$ from Proposition 2, we have that $\phi \left(p\right)$ is:

6 $$\phi \left(p\right)=\left\{\begin{array}{ccc}0& if& p>{p}_{crit}\\ 1-\frac{1}{{R}_{0}\left(1-p\right)}& if& p\le {p}_{crit}\end{array}\right.$$

For $p\le {p}_{crit}$, the probability of infection is a concave function.^4^ We can use $\phi \left(p\right)$ to characterize the equilibrium vaccination probability ${p}^{*}$. Since from Proposition 1 the equilibrium vaccination probability ${p}^{*}$ is ${\phi }^{-1}\left(\frac{{D}_{V}-{D}_{S}}{r{D}_{I}}\right)$, it follows from (6):

$$\frac{{D}_{V}-{D}_{S}}{r{D}_{I}}=1-\frac{1}{{R}_{0}\left(1-{p}^{*}\right)}\Leftrightarrow {p}^{*}=1-\frac{r{D}_{I}}{{R}_{0}\left[r{D}_{I}-{D}_{V}+{D}_{S}\right]}$$

#### Proposition 3

*The equilibrium vaccination probability* ${p}^{*}$ *is:*

7 $${p}^{*}=\left\{\begin{array}{ccc}0& if& \phi \left(0\right)\le \frac{{D}_{V}-{D}_{S}}{r{D}_{I}}\\ 1-\frac{r{D}_{I}}{{R}_{0}\left[r{D}_{I}-{D}_{V}+{D}_{S}\right]}& if& \phi \left(0\right)>\frac{{D}_{V}-{D}_{S}}{r{D}_{I}}\end{array}\right.$$

**Fig. 1:**
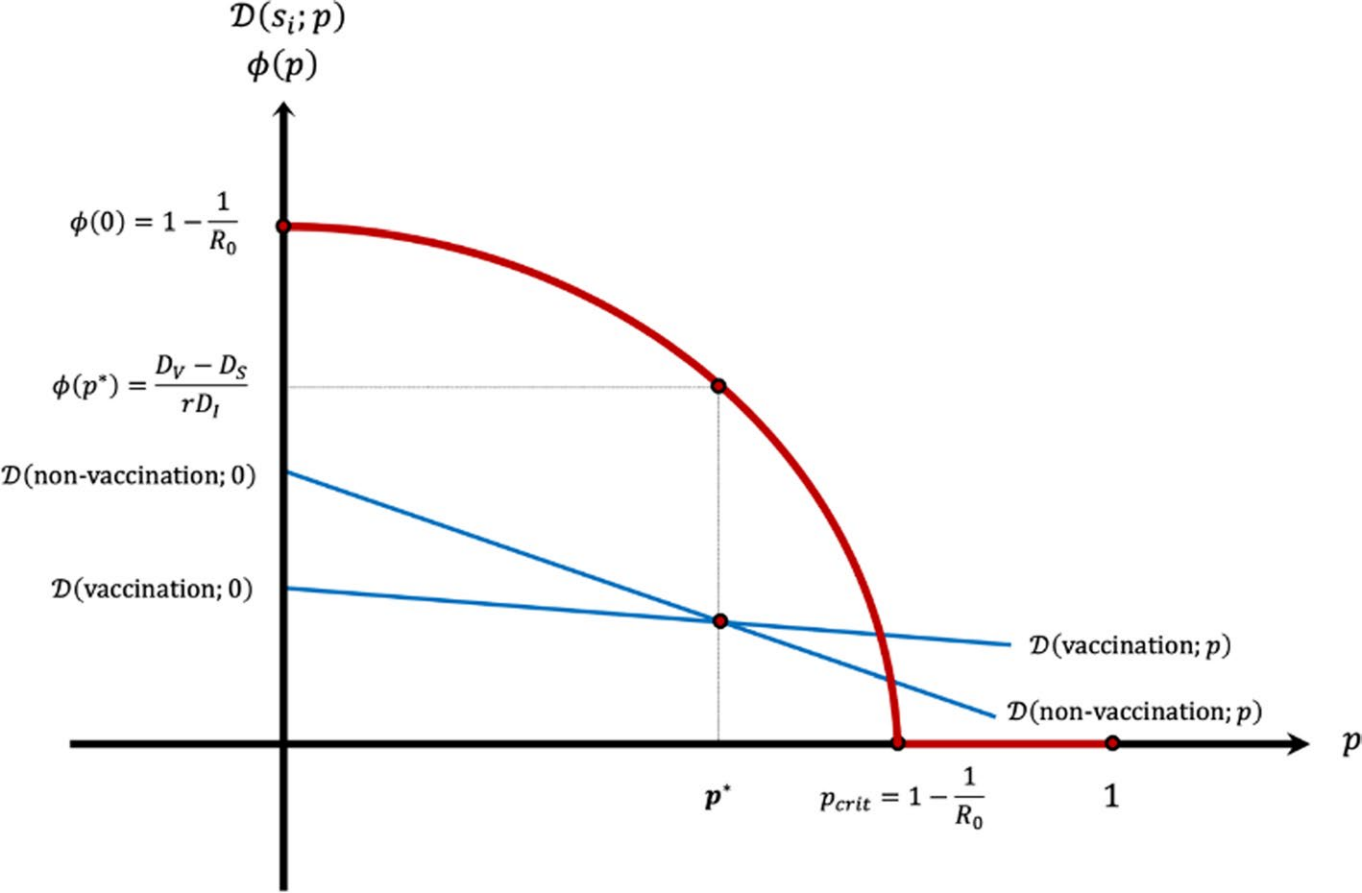
The equilibrium vaccination probability

Given the assumptions, ${p}^{*}<1.$ It is easy to see that the equilibrium vaccination probability ${p}^{*}$ increases with ${D}_{I}$, ${D}_{s}$, $r$ and ${R}_{0}$, and decreases with ${D}_{V}$.^5^ Figure 1 depicts the determination of the equilibrium vaccination probability when $\phi \left(0\right)>\frac{{D}_{V}-{D}_{S}}{r{D}_{I}}$.

## The social cost of the disease

Following Yamin and Gavious (2013), we define the social cost of the disease $SC(p)$ as the aggregate disutility of the vaccinated and unvaccinated population:

$$SC\left(p\right)=p\mathcal{D}\left(\text{vaccination; }p\right)+\left(1-p\right)\mathcal{D}\left(\text{non-vaccination; }p\right)$$

### Proposition 4

The vaccination rate that minimizes the social cost of the disease is ${p}_{crit}$, the rate that achieves herd immunity.

### Proof

See Appendix.

If it were possible to make vaccination compulsory, the population should be vaccinated until the rate compatible with herd immunity is reached. As shown in Fig. 1, the equilibrium vaccination probability ${p}^{*}$ is below the value ${p}_{crit}$. When individuals decide on vaccination, they only consider the private costs, and ignore the negative external effects of non-vaccination or the positive external effects of vaccination. As a result, the equilibrium population vaccination rate is below the social optimal rate. Since in equilibrium, $\mathcal{D}\left(\text{vaccination; }{p}^{*}\right)=\mathcal{D}\left(\text{non-vaccination;}{ p}^{*}\right)$, the social cost evaluated at the equilibrium probability ${p}^{*}$, $SC\left({p}^{*}\right)$, is equal to $\mathcal{D}\left(\text{non-vaccination; }{p}^{*}\right)= {D}_{I}\phi \left({p}^{*}\right)+{D}_{S}$. It easily follows from (6) and (7) that ${D}_{I}\phi \left({p}^{*}\right)=\frac{1}{r}[{D}_{V}-{D}_{S}]$. As $\phi \left({p}^{*}\right)=0$ when ${p}^{*}>{p}_{crit}$, we can write the equilibrium social cost as:

8 $$SC\left({p}^{*}\right)=\left\{\begin{array}{ccc}\frac{1}{r}\left[{D}_{V}-\left(1-r\right){D}_{S}\right]& if& {p}^{*}\le {p}_{crit}\\ {D}_{S}& if& {p}^{*}>{p}_{crit}\end{array}\right.$$

Since the equilibrium social cost depends on ${D}_{V}$ and ${D}_{S}$, public authority can design public policies to modify such parameters to promote vaccination and reduce the social cost of the disease.^6^ In this regard, the public authority may adopt measures to reduce the costs of vaccination ${D}_{V}$, promoting confidence in the safety and efficacy of vaccines, refuting wrong and biased information provided by vaccine deniers, and raising social responsibility. All these measures can be integrated into what we refer to as informational campaigns. Likewise, the public authority can adopt measures to modify the behavior of individuals without necessarily changing their minds about vaccines. Within this group of measures, we may consider, among others, the requirement of vaccination certificates for access to some venues (*e. g.,* restaurants) or for traveling that affect ${D}_{S}$. These measures increase the disutility of non-vaccination.

Next, we analyse each type of intervention separately, and characterize the optimal policies. From now on, we model the interaction between the public authority and the individuals as a two-stage game. In the first stage, the public authority chooses the intervention policy to minimize the total cost of the disease (*i.e.,* the social cost plus the cost of the policy); in the second stage, the individuals, after observing the policy, make their decision on vaccination. We use as equilibrium concept the subgame perfect equilibrium.

### Informational campaign

Let $x\ge 0$ denote the level or intensity of the informational campaign, and $C\left(x\right)$ its cost, with ${C}{\prime}\left(x\right)>0$ and ${C}^{{\prime}{\prime}}\left(x\right)\ge 0$. The perceived disutility when vaccinated is ${D}_{V}\left(x\right)$, with ${D}_{V}\left(0\right)={D}_{V}$, ${D}_{V}{\prime}\left(x\right)<0$ and ${D}_{V}{\prime}{\prime}(x)\ge 0$ $\forall x\ge 0$. The higher the intensity of the campaign, the lower the disutility when vaccinated. Given $x$, the individuals choose the vaccination probability ${p}^{*}(x)$. Notice that the level of the informational campaign cannot induce an equilibrium vaccination probability above the critical probability ${p}_{crit}$, as it is costly and from (8), it has no effect on the social cost given the eradication of the disease. Therefore, the level of the informational campaign is bounded above by a value $\overline{x}$ such that ${p}^{*}\left(\overline{x}\right)={p}_{crit}$. From (7) it follows that $\overline{x}$ is defined by ${D}_{V}(\overline{x})={D}_{S}$. The public authority chooses $x\in [0,\overline{x}]$ to minimize the total cost $CT\left(x\right)=SC\left({p}^{*}\left(x\right)\right)+ C\left(x\right)$. From (8), the public authority solves:

$$\underset{x\in [0, \overline{x}]}{{\text{min}}}CT\left(x\right)=\frac{1}{r}[{D}_{V}(x)-\left(1-r\right){D}_{S}]+C(x)$$

As the social cost decreases with $x$ and the policy cost is increasing in $x$, it follows that the solution ${x}^{*}$ is either interior ($0<{x}^{*}< \overline{x})$ or corner (${x}^{*}= \overline{x})$. The first order condition is

$$\frac{dCT(x)}{dx}=0\Leftrightarrow {D}_{V}{\prime}\left(x\right)+rC{\prime}(x)=0$$

The interior solution satisfies this condition, and for the corner solution, $\frac{dCT(x)}{dx}<0$. Notice that ${\left.\frac{dCT\left(x\right)}{dx}\right|}_{x=0}<0$. Let ${x}^{*}$ such that $-{D}_{V}{\prime}\left({x}^{*}\right)=rC{\prime}({x}^{*})$. If ${x}^{*}<\overline{x}$, then ${x}^{*}$ is the solution to the problem. Otherwise, the solution is $\overline{x}$. Notice that the second order condition ($\frac{{d}^{2}CT(x)}{d{x}^{2}}>0)$ is satisfied given the assumptions on ${D}_{V}\left(x\right)$ and $C\left(x\right)$. An alternative form to express the solution to the problem is:

$$If\;D_{V}{\prime} \left( {\overline{x}} \right) + rC^{\prime}\left( {\overline{x}} \right) \le 0 \Rightarrow x^{*} = \overline{x} and p = p_{crit}$$

$${\text{If}}\;D_{V}{\prime} \left( {\overline{x}} \right) + rC^{\prime}\left( {\overline{x}} \right) > 0 \Rightarrow x^{*} < \overline{x} and p < p_{crit}$$

It does not necessarily follow that it is optimal to reach herd immunity when the provision of incentives to get vaccinated is costly. Intuitively, when the cost of the intervention is relatively high, herd immunity is not reached, and the system converges to a stationary state in which the disease in endemic. However, the vaccination rate is higher than the rate without intervention. If the ratio between the marginal reduction of ${D}_{V}$ and the marginal cost of the intervention evaluated at the highest feasible value for the intensity of the campaign is higher that the effectiveness of the vaccine, then the optimal level of the intervention is the highest feasible value and herd immunity is reached. Otherwise, the intensity of the campaign is below its highest feasible value and the vaccination rate is below than the level of herd immunity.

#### Proposition 5

When the health authority uses only an informational campaign to incentive vaccination, herd immunity is reached if and only if ${D}_{V}^{\mathrm{^{\prime}}}\left(\overline{x}\right)+r{C}^{\mathrm{^{\prime}}}\left(\overline{x}\right)\le 0$, where $\overline{x}$ satisfies ${D}_{V}(\overline{x})={D}_{S}$. Otherwise, the optimal campaign increases the vaccination rate without reaching herd immunity.

### Policy to increase the disutility of non-vaccination

Let $y\ge 0$ denote the level or intensity of the sanctions or restrictions the health authority may impose on the non-vaccinated individuals, and $H\left(y\right)$ their cost, with ${H}{\prime}\left(y\right)>0$ and $H{\prime}{\prime}(y)\ge 0$.

The perceived disutility from non-vaccination is ${D}_{S}\left(y\right)$, with ${D}_{S}\left(0\right)={D}_{S}$, ${D}_{S}{\prime}\left(y\right)>0$ and ${D}_{S}{\prime}{\prime}\le 0$ $\forall y>0$. The higher the intensity of the restrictions, the greater the costs from non-vaccination. As before, given $y$, the individuals decide the vaccination probability ${p}^{*}(y)$. The intensity $y$ is bounded above by the value $\overline{y }$ such that ${p}^{*}\left(\overline{y }\right)={p}_{crit}$. From (7), $\overline{y }$ is defined as ${D}_{S}\left(\overline{y }\right)={D}_{V}$.The public authority chooses $y\in [0,\overline{y }]$ to minimize the total cost $CT\left(y\right)=SC\left( {p}^{*}\left(y\right)\right)+H(y)$. From (8), the public authority solves:

$$\underset{y\ge \left[0, \overline{y}\right]}{{\text{min}}}CT\left(y\right)=\frac{1}{r}\left[{D}_{V}-\left(1-r\right){D}_{S}\left(y\right)\right]+H(y)$$

The first order condition is:

$$\frac{dCT\left( y \right)}{{dy}} = 0 \Leftrightarrow - \left( {1 - r} \right)D_{S}{\prime} \left( y \right) + rH^{\prime}\left( y \right) = 0$$

The interior solution satisfies this condition, and the corner solution satisfies $\frac{dCT(y)}{dy}<0$. Notice that ${\left.\frac{dCT\left(y\right)}{dy}\right|}_{y=0}<0$. Let ${y}^{*}$ be such that $(1-r){D}_{S}{\prime}{y}^{*}=rH{\prime}({y}^{*})$. If ${y}^{*}<\overline{y}$, then ${y}^{*}$ is the solution. Otherwise, the solution $\overline{y}$. Notice that the second order condition ($\frac{{d}^{2}CT(y)}{d{y}^{2}}>0)$ is satisfied given the assumption on ${D}_{S}\left(y\right)$ y $H(y)$. An alternative form to express the solution is:

$$If \left( {1 - r} \right)D_{S}{\prime} \left( {\overline{y}} \right) \ge rH^{\prime}\left( {\overline{y}} \right) \Rightarrow y^{*} = \overline{y} and p = p_{crit}$$

$$If \left( {1 - r} \right)D_{S}{\prime} \left( {\overline{y}} \right) < rH^{\prime}\left( {\overline{y}} \right) \Rightarrow y^{*} < \overline{y} and p < p_{crit}$$

As before, when the cost of the intervention is relatively high, herd immunity is not reached, and the system converges to the stationary state with an endemic disease.

#### Proposition 6

When the public authority uses only measures to increase the disutility for non-vaccination, herd immunity is reached if and only if $(1-r){D}_{S}^{\mathrm{^{\prime}}}\left(\overline{y }\right)\ge r{H}^{\mathrm{^{\prime}}}\left(\overline{y }\right)$, where $\overline{y}$ satisfies ${D}_{s}(\overline{y})={D}_{S}$. Otherwise, the optimal intervention policy increases the vaccination rate without reaching herd immunity.

### Monetary incentives

Public authorities may also consider monetary incentives (*i.e.,* paying a sum of money $m$ to individuals who get vaccinated)) to incentive vaccination.^7^ If the health authority pays $m\ge 0$ to each vaccinated individual, the expected disutility or cost if vaccinated when the population vaccination rate is $p$ is given by $\mathcal{D}\left(\text{vaccination; }p\right)={D}_{V}+\left(1-r\right)\phi \left(p\right){D}_{I}-m$. From the analysis in Sect. "The model", it follows that the equilibrium vaccination probability ${p}^{*}$ is $1-\frac{r{D}_{I}}{{R}_{0}\left[r{D}_{I}-{D}_{V}+{D}_{S}+m\right]}$ if $\phi \left(0\right)>\frac{{D}_{V}-{D}_{S}-m}{r{D}_{I}}$. The social cost of the disease $SC({p}^{*})$ from Eq. (8) is now:

9 $$SC\left( {p^{*} } \right) = \left\{ {\begin{array}{*{20}l} {\frac{1}{r}\left[ {D_{V} - \left( {1 - r} \right)D_{S} - m} \right]} \hfill & {if\;p^{*} \le p_{crit} } \hfill \\ {D_{S} } \hfill & {if\;p^{*} > p_{crit} } \hfill \\ \end{array} } \right.$$

As before, in the first stage of the game, the health authority chooses $m$ to minimize the total cost, and in the second stage, given $m$, the individuals decide the vaccination probability ${p}^{*}(m)$. The monetary payment is bounded above by the value $\overline{m }$ such that ${p}^{*}(m){=p}_{crit}$. Thus, $\overline{m }={D}_{V}-{D}_{S}$.

The health authority chooses $m\in [0,\overline{m }]$ to minimize the total cost $CT\left(m\right)=SC\left({p}^{*}\left(m\right)\right)+ {p}^{*}\left(m\right) m$. In this case, the cost of the intervention is the amount paid to each vaccinated individual multiplied by the proportion of the population that is vaccinated in equilibrium: ${p}^{*}\left(m\right)m$. From (9), the health authority solves:

$$\underset{m\in \left[0,\overline{m } \right]}{{\text{min}}}CT\left(m\right)=\frac{1}{r}\left[{D}_{V}-\left(1-r\right){D}_{S}-m\right]+ {p}^{*}(m)m$$

It follows that^8^:

$$\frac{dCT(m)}{dm}=-\frac{1}{r}+ {p}^{*}\left(m\right)+m\frac{d{p}^{*}\left(m\right)}{dm}=-\frac{1-r}{r}-\frac{r{D}_{I}\left(r{D}_{I}-{D}_{V}+{D}_{S}\right)}{{R}_{o}{\left[r{D}_{I}-{D}_{V}+{D}_{S}+m\right]}^{2}}<0$$

as we are assuming $r{D}_{I}>{D}_{V}-{D}_{S}$. As the total costs decrease with $m$, we have that the optimal monetary incentive must be the maximum one, and herd immunity is achieved: ${m}^{*}=\overline{m }$ y $p={p}_{crit}$.

#### Proposition 7

Herd immunity is achieved when the public authority gives a monetary payment to vaccinated individuals.

## The optimal policy to promote vaccination

In real world, the public authority is not restricted to use only one policy to promote vaccination, and may use all the instruments at its disposal. In this section, we assume that the three types of intervention previously analyzed are now available. Let $CT\left(x,m,y\right)$ denote the total cost of the disease when the public policy is $(x,m, y)$. We keep on using the same notation for the intervention policies as well as their definitions. Considering the equilibrium social cost given in (9), the public authority solves:

$$\begin{gathered} \mathop {\min }\limits_{x,m,y} CT\left( {x,m,y} \right) = SC({ }p^{*} \left( {x,y, m} \right)) + C\left( x \right) + H\left( y \right) + { }p^{*} \left( {x,m,y} \right) m \hfill \\ \quad \quad \quad \quad \quad \quad \quad = { }\frac{1}{r}[D_{V} \left( x \right) - \left( {1 - r} \right)D_{S} \left( y \right) - m] + C\left( x \right) + H\left( y \right) + p^{*} \left( {x,m,y} \right) m \hfill \\ {\text{s.a.}}\;x \ge 0,\;m \ge 0,\;y \ge 0 \hfill \\ p^{*} \left( {x,m,y} \right) = 1 - \frac{{rD_{I} }}{{R_{0} \left[ {rD_{I} - D_{V} \left( x \right) + D_{S } \left( y \right) + m} \right]}} \hfill \\ p^{*} \left( {x,m,y} \right) \le p_{crit} \hfill \\ \end{gathered}$$

The optimal public policy cannot induce an equilibrium vaccination probability above the critical probability ${p}_{crit}$ as the public authority would not be minimizing the total cost. In the solution to the problem, the vaccination probability must be equal to the critical probability ${p}_{crit}$ that guarantees herd immunity, and therefore, the last constraint of the problem must be binding. Otherwise, it would be possible to find another public policy with a lower total cost. Note that the total cost is decreasing in the monetary incentive:

$$\frac{\partial CT}{\partial m}=-\frac{1}{r}+{p}^{*}\left(x,m,y\right)+m\frac{\partial {p}^{*}}{\partial m}=1-\frac{1}{r}-\frac{r{D}_{I}\left[r{D}_{I}-{D}_{V}\left(x\right)+{D}_{S }\left(y\right)\right]}{{R}_{0}{\left[r{D}_{I}-{D}_{V}\left(x\right)+{D}_{S }\left(y\right)+m\right]}^{2}}<0$$

If the equilibrium vaccination probability were lower than the critical probability ${p}_{crit}$, the public authority could increase the monetary incentive without violating any constraint, and the total cost would be reduced. Therefore, the last constraint must be binding: ${p}^{*}\left(x,m,y\right)={p}_{crit}$. Considering the expressions of both probabilities, it follows that ${D}_{V}\left(x\right)-m={D}_{S}(y)$.

The problem can now be rewritten as:

$$\begin{gathered} \mathop {\min }\limits_{x,m,y} CT\left( {x,m,y} \right) = \frac{1}{r}[D_{V} \left( x \right) - \left( {1 - r} \right)D_{S} \left( y \right) - m] + C\left( x \right) + H\left( y \right) + p_{crit} m \hfill \\ {\text{s.a.}}{\text{}}{}\;x \ge 0,\;m \ge 0,\;y \ge 0 \hfill \\ \end{gathered}$$

If we replace $m$ with ${D}_{V}\left(x\right)-{D}_{S}(y)$ in the objective function, we have:

$$\begin{gathered} \mathop {\min }\limits_{x,y} CT\left( {x,y} \right) = D_{S} \left( y \right) + C\left( x \right) + H\left( y \right) + p_{crit} \left( {D_{V} \left( x \right) - D_{S} \left( y \right)} \right) \hfill \\ {\text{s.a.}}\;x \ge 0, y \ge 0 \hfill \\ \end{gathered}$$

As the objective function grows with$y$,^9^ we must have in the solution ${y}^{*}=0$, and ${D}_{S}\left(0\right)={D}_{S}.$ The public authority will not use measures to increase the cost of non-vaccination. Let $\overline{x}$ be the level of the informational campaign defined by ${D}_{V}\left(\overline{x}\right)={D}_{S}$. Note that $\overline{x}$ is the level of the campaign that satisfies the constraint $m={D}_{V}\left(x\right)-{D}_{S}$ for $m=0$. Therefore, the range of $x$ es $[0,\overline{x}$] and that of $m$ is $[0,{D}_{V}-{D}_{S}]$. The problem can be rewritten as follows:

$$\begin{gathered} \mathop {\min }\limits_{x} CT\left( x \right) = p_{crit} D_{V} \left( x \right) + \left( {1 - p_{crit} } \right)D_{S} + C\left( x \right) \hfill \\ {\text{s.a.}}\;x \in \left[ {0, \overline{x}{ }} \right] \hfill \\ \end{gathered}$$

The derivative of the objective function with respect to $x$ is $\frac{dCT(x)}{dx}= {p}_{crit}{D}_{V}{\prime}\left(x\right)+C{\prime}(x)$. Suppose that there exists a value ${x}^{*}\in (0,\overline{x})$ such that ${p}_{crit}{D}_{V}{\prime}\left({x}^{*}\right)+{C}{\prime}\left({x}^{*}\right)=0$, *i.e.,* ${x}^{*}$ satisfies the first order condition for an interior solution. Then, ${x}^{*}$ would be the optimal level for the informational campaign. The optimal monetary incentive would be ${m}^{*}={D}_{V}\left({x}^{*}\right)-{D}_{S}$. A necessary and sufficient condition for this interior solution is ${p}_{crit}{D}_{V}{\prime}\left(\overline{x}\right)+{C}{\prime}\left(\overline{x}\right)>0$. If ${p}_{crit}{D}_{V}{\prime}\left(\overline{x}\right)+{C}{\prime}\left(\overline{x}\right)\le 0$, the objective function decreases with $x$, and the solution to the problem is $\overline{x}$. In this case, the public authority does not use the monetary incentive: ${m}^{*}=0$. Note that the sufficient condition for the minimization problem ($\frac{{d}^{2}CT(x)}{d{x}^{2}}>0)$ is satisfied.

### Proposition 8

If ${p}_{crit}{D}_{V}^{\mathrm{^{\prime}}}\left(\overline{x}\right)+{C}^{\mathrm{^{\prime}}}\left(\overline{x}\right)>0$, where $\overline{x}$ satisfies ${D}_{V}\left(\overline{x} \right)={D}_{S}$, the optimal intervention public policy consists of both monetary incentives and an informational campaign. The intensity of the campaign is ${x}^{*}<\overline{x}$, where ${x}^{*}$ satisfies ${p}_{crit}{D}_{V}^{\mathrm{^{\prime}}}\left({x}^{*}\right)+C\mathrm{^{\prime}}({x}^{*})=0$, and the monetary incentive is ${m}^{*}={D}_{V}\left({x}^{*}\right)-{D}_{S}$. If ${p}_{crit}{D}_{V}^{\mathrm{^{\prime}}}\left(\overline{x}\right)+{C}^{\mathrm{^{\prime}}}\left(\overline{x}\right)\le 0$, the optimal intervention public policy does not include monetary incentives. The optimal level of the campaign is $\overline{x}$.

Intuitively, the result is driven by the distinct nature of the two types of interventions. On the one hand, the benefits of an informational campaign have the characteristics of a public good: *non-rivalry and non-excludability.* The benefits of the campaign reach the entire population and the positive effects enjoyed by one individual do not reduce the benefits available to others. On the contrary, the monetary incentives have the characteristics of private goods (*rivalry and excludability*). The money received by a vaccinated individual is not available to another individual, and non-vaccinated individuals are excluded unless they decide to get vaccinated. If the informational campaign is effective in the sense that it convincingly reaches the majority of the population, the public authority will prefer to use this type of intervention rather than one based on monetary incentives, since it achieves the same goal (herd immunity) while spending less. Analytically, this is equivalent to saying that the marginal effect of the campaign on the perceived vaccination costs is greater than its marginal cost for all values of $x$. However, if the informational campaign is not sufficiently effective, the public authority will choose the level of intensity for which the marginal effect on perceived vaccination costs equals the marginal cost of the campaign, and the public intervention will be complemented with monetary incentives to achieve a vaccination level that will eradicate the disease. In this case, the campaign is less effective than the monetary incentives beyond a level ${x}^{*}<\overline{x }$. In the previous section, we have shown then herd immunity could be reached when the public authority used only monetary incentives. We have seen now that such immunity is also achieved when the public authority is not constrained to use only monetary incentives. Our result shows that it is too costly to use only monetary incentives. Therefore, in the optimal policy, these incentives are either combined with an informational campaign, or even not used at all. If they are used, vaccinated individuals are paid less than in the case in which the public authority uses only monetary incentives. Surprisingly, when there are no restrictions to the instruments the public authority can use to increase the probability of vaccination, the use of measures to increase the disutility of the non-vaccinated are not part of the optimal policy.

**Fig. 2:**
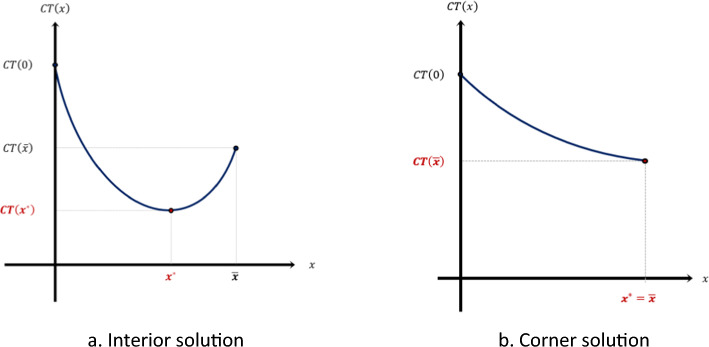
The optimal intensity of the informational campaign

Figure 2a and b depict the solution stated in Proposition 8. In Figure 2a, we see that the optimal level of the informational campaign is lower than $\overline{x }$. The function $CT\left(x\right)$ is U-shaped. In Figure 2b, the total cost function is decreasing in $x$, and therefore, the intensity of the informational campaign that minimizes the total cost is the maximum value $\overline{x}$. We include in the appendix a graphical analysis of the optimal policy in the $x-m$ plane.

It can be shown that the interior solution ${x}^{*}$ grows with ${p}_{crit}$. If we totally differentiate the first order condition of the minimization problem:

$$p_{crit} D_{V}^{^{\prime\prime}} \left( x \right)dx^{*} + D_{V}{\prime} \left( x \right)dp_{crit} + C^{\prime\prime}\left( x \right)dx^{*} = 0 \Rightarrow \frac{{dx^{*} }}{{dp_{crit} }} = \frac{{ - D_{V} ^{\prime}\left( x \right)}}{{p_{crit} D_{V}^{^{\prime\prime}} \left( x \right) + C^{\prime\prime}\left( x \right)}} > 0$$

Since the denominator is positive for the second order condition. The higher the critical probability that guarantees herd immunity, the higher the intensity of the informational campaign, and the lower the monetary incentive.

## Conclusions

In this paper, we have focused on the economics of vaccination, and we have characterized the optimal policy that can be used to raise the vaccination rate to eradicate a generic infectious disease in a context where vaccination suffers from a *free-riding* problem. In particular, we have considered three types of public policies defined by measures that affect the costs of vaccination and non-vaccination. Measures based on informational campaigns and on monetary incentives seek to incentivize vaccination, while policies based on restrictions affecting the costs of non-vaccination try to make non-vaccination less attractive. We have analyzed the optimality of each policy at a time, and the optimal policy when the public authority can use all the instruments available and it is not restricted to use only one type of policy. We have found that the optimal policy must include, at least, some expenditure in informational campaigns, together with the provision of monetary incentives, when the efficacy of the campaign is not high enough to eradicate the disease. On the other hand, the optimal policy may consist only of an informational campaign when its efficacy is sufficiently high. Measures based on restrictions against non-vaccinated individuals are not used in the optimal policy, as they increase the total cost without increasing the vaccination rate.

Some of the implications of the analysis are applicable to the COVID-19 pandemic. We have modeled, for a generic disease, the steady state to which the system converges.

The disease would be eradicated if herd immunity is achieved. We should expect the same for COVID-19. However, it appears that COVID-19 will become an endemic disease, as is the case in our model with the generic disease in one of the steady states. We have seen that we can increase the vaccination rate with public policies. Although the vaccine against COVID-19 appears to be effective, it does not provide lifelong immunity, and vaccination might be required regularly as, for instance, it happens with the seasonal flu. In this context, the model prescribes the use of informational campaigns to promote vaccination, and perhaps, informational campaigns combine with monetary incentives.

The model we have used is highly stylized and has some limitations. We have considered a population of identical individuals. In the real world, it is well known that each person values differently the risks of vaccination or infection. For example, the costs of non-vaccination are higher for socially committed individuals as they consider the negative external effects of non-vaccination. Also, individuals may derive disutility from non-vaccination if there is a social norm making non-vaccination unacceptable. Social pressure to influence socially well-seen behaviors may explain why some countries have higher vaccination rates. An extension of the model could introduce individual heterogeneity, and index individuals according to the degree to which they internalize the external effects of vaccination or are influenced by social norms. Vaccination probabilities would differ across individuals, with the population vaccination rate being the average of the individual probabilities. We leave this extension for future research.

## Figures and Tables

**Fig. 3:**
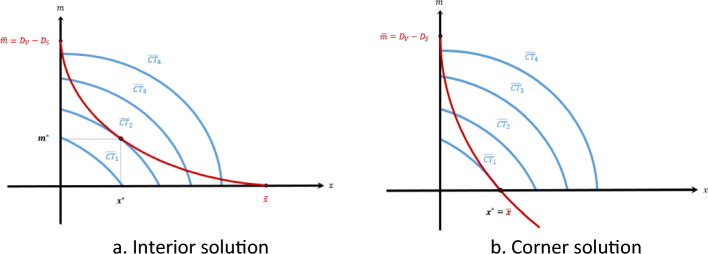
The optimal policy

## Appendix

### Proof of Proposition 1

If the individual gets vaccinated, $\phi \left(1\right)=0$. The individual gets vaccinated if $\mathcal{D}\left(\text{vaccination; }1\right)$ is lower $\mathcal{D}\left(\text{non-vaccination; }1\right)$, or if ${D}_{V}<{D}_{S}$. As this condition, given the assumptions, is not satisfied, it follows that either the individual does not get vaccinated or he/she vaccinates with a certain probability. The individual does not get vaccinated if $\mathcal{D}\left(\text{non-vaccination; }0\right)\le \mathcal{D}\left(\text{vaccination; }0\right)$. Thus, it is required $r\phi \left(0\right){D}_{I}\le {D}_{V}-{D}_{S}$, or $\phi \left(0\right)\le \frac{{D}_{V}-{D}_{S}}{r{D}_{I}}$. If this condition is not satisfied, the individual will get vaccinated with a probability ${p}^{*}$ for which he/she is indifferent between vaccination and non-vaccination:

$$\begin{gathered} {\mathcal{D}}\left( {{\text{vaccination}};p^{*} } \right) = {\mathcal{D}}\left( {{\text{non - vaccination}};p^{*} } \right) \\ \Updownarrow \\ D_{V} + \left( {1 - r} \right)\phi \left( {p^{*} } \right)D_{I} = \phi \left( {p^{*} } \right)D_{I} + D_{S} \Leftrightarrow \phi \left( {p^{*} } \right) = \frac{{D_{V} - D_{S} }}{{rD_{I} }} \\ \end{gathered}$$

Therefore, the equilibrium vaccination probability ${p}^{*}$ is defined by ${\phi }^{-1}\left(\frac{{D}_{V}-{D}_{S}}{r{D}_{I}}\right)$. (Q.E.D.)

### Proof of Proposition 2

In the steady state, the rates of change of the susceptible and infected subgroups are 0. From (5), it follows that $\frac{dI}{dz}=\left(1+f\right)I\left[{R}_{0}S-1\right]=0$. Since $\left(1+f\right)$ is strictly positive, the equation is only satisfied when $I=0$ or ${R}_{0}S-1=0$. Therefore, there may be two steady states. In one of them, the disease is eradicated and there are no infected individuals: $I=0$. From (4), it follows that $\frac{dS}{{dz}} = f\left( {1 - p - S} \right) = 0 \Rightarrow S = 1 - p$. In the other steady state, the disease becomes endemic: $R_{0} S - 1 = 0 \Leftrightarrow S = \frac{1}{{R_{0} }}$. From (4), we have: $\frac{dS}{{dz}} = { }f\left( {1 - p} \right) - R_{0} \left( {1 + f} \right)\frac{1}{{R_{0} }}I - f\frac{1}{{R_{0} }} = 0 \Leftrightarrow { }I = \frac{{f\left[ {R_{0} \left( {1 - p} \right) - 1} \right]}}{{R_{0} \left( {1 + f} \right)}}$. Since we need $I>0$ for the disease to be endemic, we require ${R}_{0}\left(1-p\right)-1>0$, or $p<1-\frac{1}{{R}_{0}}={p}_{crit}$. (Q.E.D.)

### Proof of Proposition 4

The social cost of the disease is defined as $p\mathcal{D}\left(\text{vaccination; }p\right)+\left(1-p\right)\mathcal{D}\left(\text{non-vaccination; }p\right)$. By considering the expressions for $\mathcal{D}\left(\text{vaccination; }p\right)$ and $\mathcal{D}\left(\text{non-vaccination; }p\right)$ from section "The vaccination game", we can write:

$$\begin{aligned} SC\left( p \right) = & p\left[ {D_{V} + \left( {1 - r} \right)\phi \left( p \right)D_{I} } \right] + \left( {1 - p} \right)\left[ {\phi \left( p \right)D_{I} + D_{S} } \right] \\ = & pD_{V} + \left( {1 - p} \right)D_{S} + \phi \left( p \right)D_{I} \left( {1 - pr} \right) \\ \end{aligned}$$

The derivative of $SC\left(p\right)$ with respect to $p$ yields:

$$\frac{dSC}{dp}={D}_{V}-{D}_{S}-r{D}_{I}\phi \left(p\right)+{D}_{I}\left(1-pr\right)\frac{d\phi \left(p\right)}{dp}$$

After replacing $\phi \left(p\right)$ with its expression form (6) and $\frac{d\phi \left(p\right)}{dp}$ with $-\frac{1}{{R}_{0}{\left(1-p\right)}^{2}}$, we get:

$$\frac{dSC}{dp}={D}_{V}-{D}_{S}-r{D}_{I}\left[1-\frac{1}{{R}_{0}\left(1-p\right)}\right]-{D}_{I}\left(1-pr\right)\left[\frac{1}{{R}_{0}{\left(1-p\right)}^{2}}\right]={D}_{V}- {D}_{S}-r{D}_{I}-\frac{{D}_{I}(1-r)}{{R}_{0}{\left(1-p\right)}^{2}}$$

This derivative is negative since we are assuming that $r{D}_{I}>{D}_{V}-{D}_{S}$. Therefore, the social cost decreases with $p$. Hence, from a social perspective, the optimal vaccination rate must be ${p}_{crit}$. (Q.E.D.)

## Graphical analysis of the optimal policy in the ${\varvec{x}}-{\varvec{m}}$ plane

The problem to choose the optimal policy could have been written as:

$$\begin{gathered} \mathop {\min }\limits_{x,m} CT\left( {x,m} \right) = p_{crit} m + D_{S} + C\left( x \right) \hfill \\ {\text{s}}{\text{. a}}{.}\;x \in \left[ {0, \overline{x} } \right],\;m \in [0,D_{V} - D_{S} ] \hfill \\ D_{V} \left( x \right) - m = D_{S} \hfill \\ \end{gathered}$$

In the $x-m$ plane, the equality constraint is decreasing and convex. If we totally differentiate it, we have:

$$D_{V}{\prime} \left( x \right)dx - dm = 0 \Rightarrow \frac{dm}{{dx}} = D_{V}{\prime} \left( x \right)\left\langle {0, \frac{{d^{2} m}}{{dx^{2} }} = D_{V}^{^{\prime\prime}} \left( x \right)} \right\rangle 0$$

In the context of the problem, an isocost curve of level $\overline{CT}$ is the set of combinations $(x,m)$ such that the objective function takes the value $\overline{CT}$. Formally, ${ISO}_{\overline{CT}}=\left\{\left(x,m\right) | {p}_{crit}m+{D}_{S}+C\left(x\right)=\overline{CT}\right\}$. The farther the isocost is from the origin, the higher the total cost. The isocost curves are decreasing and concave. If we totally differentiate the equation of the isocost curve, we have:

$$p_{crit} dm + C\prime \left( x \right)dx = 0 \Rightarrow \frac{dm}{{dx}} = - \frac{C\prime \left( x \right)}{{p_{crit} }} < 0, \frac{{d^{2} m}}{{dx^{2} }} = - \frac{C\prime \prime \left( x \right)}{{p_{crit} }} < 0$$

In the interior solution of the problem, the slope of the equality constraint, in absolute value, equals the slope of the isocost curve. In other words, the interior solution $$({x}^{*},{m}^{*})$$ is a tangency point: $$-{D}_{V}^{\mathrm{^{\prime}}}\left({x}^{*}\right)=\frac{{C}^{\mathrm{^{\prime}}}{x}^{*}}{{p}_{crit}}$$. In the corner solution $$(\overline{x}$$,$$0)$$, the slope of the equality constraint, in absolute value, is bigger than the slope of the isocost curve: $$-{D}_{V}^{\mathrm{^{\prime}}}\overline{x}>\frac{{C}^{\mathrm{^{\prime}}}(\overline{x})}{{p}_{crit}}$$. Figures 3a and b depict both solutions. In Fig. 3a, the tangency point between the constraint and the isocost curve illustrates the solution to the problem. In Fig. 3b, there is no tangency, and we have a corner solution.

## Footnotes

1 Vaccination costs include, among others, the value of the time required to go to the vaccination center, the pain of the syringe, and the adverse effects of the vaccine. Non-vaccination costs include the cost related to the symptoms of the disease and to possible hospitalizations and the cost individual bears from the restrictions that public authorities may impose on unvaccinated individual.

2 See also Francis (1997) who derives the same result in a continuous-time dynamic model.

3 $$\frac{dS}{dz}=\frac{dS}{dt}\frac{dt}{dz}$$ and $$\frac{dI}{dz}=\frac{dI}{dt}\frac{dt}{dz}$$, where $$\frac{dt}{dz}=1/\gamma$$.

4 $$\frac{d\phi (p)}{dp}=-\frac{1}{{R}_{0}{\left(1-p\right)}^{2}}<0$$ and $$\frac{{d}^{2}\phi (p)}{d{p}^{2}}=-\frac{2}{{R}_{0}{\left(1-p\right)}^{3}}<0$$.

5 $$\frac{\partial {p}^{*}}{\partial {D}_{V}}=\frac{-r{D}_{I}}{{R}_{0}{\left(r{D}_{I}-{D}_{V}+{D}_{S}\right)}^{2}}<0; \frac{\partial {p}^{*}}{\partial {D}_{S}}=\frac{{R}_{0}r{D}_{I}}{{R}_{0}{\left(r{D}_{I}-{D}_{V}+{D}_{S}\right)}^{2}}>0; \frac{\partial {p}^{*}}{\partial {D}_{I}}=\frac{r\left({D}_{V}-{D}_{S}\right)}{{R}_{0}{\left(r{D}_{I}-{D}_{V}+{D}_{S}\right)}^{2}}>0; \frac{\partial {p}^{*}}{\partial r}=\frac{{D}_{I}\left({D}_{V}-{D}_{S}\right)}{{R}_{0}{\left(r{D}_{I}-{D}_{V}+{D}_{S}\right)}^{2}}>0; \frac{\partial {p}^{*}}{\partial {R}_{0}}=\frac{r{D}_{I}}{{R}_{0}^{2}\left(r{D}_{I}-{D}_{V}+{D}_{S}\right)}>0$$.

6 Note that, since the equilibrium social cost does not depend on ${D}_{I}$, it would not make sense to adopt any measure affecting risk perception or the costs from infection.

7 Fining non-vaccinated has been considered in some countries (see, for example, Vogel and Duong (2022) for a discussion of this intervention applied to COVID-19 in Canada). In Europe, Austria and Greece have used fines to incentive COVID-19 vaccination. As stated by Vogel and Duong, this issue raises ethical considerations, and fines may excessively penalize poorer individuals. It is thought that financial penalties should be last resort to promote vaccination. We have not included fines in our analysis due to their low utilization in real world. Nevertheless, we think that modelling the use of fines for the non-vaccinated would be similar to the analysis of the monetary payments to the vaccinated. In the context of our model, the public authority would use the highest available fine compatible with achieving herd immunity. The analysis is available upon request.

8 Notice that $$\frac{d{p}^{*}\left(m\right)}{dm}=\frac{r{D}_{I}}{{R}_{o}{\left[r{D}_{I}-{D}_{V}+{D}_{S}+m\right]}^{2}}$$.

9 $$\frac{\partial CT}{\partial y}={D}_{S}{\prime}\left(y\right)\left(1-{p}_{crit}\right)+H{\prime}(y)>0$$.
